# Using a multiwavelength suite of microwave instruments to investigate the microphysical structure of deep convective cores

**DOI:** 10.1002/2016JD025269

**Published:** 2016-08-24

**Authors:** A. Battaglia, K. Mroz, Tim Lang, F. Tridon, S. Tanelli, Lin Tian, Gerald M. Heymsfield

**Affiliations:** ^1^ National Center Earth ObservationUniversity of LeicesterLeicesterUnited‐Kingdom; ^2^ Earth Observation Science, Department of Physics and AstronomyUniversity of LeicesterLeicesterUK; ^3^ NASA Marshall Space Flight CenterHuntsvilleAlabamaUSA; ^4^ Jet Propulsion LaboratoryCalifornia Institute of TechnologyPasadenaCaliforniaUSA; ^5^ NASA Goddard Space Flight CenterGreenbeltMarylandUSA; ^6^ Goddard Earth Sciences Technology and Research ProgramMorgan State UniversityBaltimoreMarylandUSA

**Keywords:** radar, microphysics, convection, retrieval

## Abstract

Due to the large natural variability of its microphysical properties, the characterization of solid precipitation is a longstanding problem. Since in situ observations are unavailable in severe convective systems, innovative remote sensing retrievals are needed to extend our understanding of such systems. This study presents a novel technique able to retrieve the density, mass, and effective diameter of graupel and hail in severe convection through the combination of airborne microwave remote sensing instruments. The retrieval is applied to measure solid precipitation properties within two convective cells observed on 23–24 May 2014 over North Carolina during the IPHEx campaign by the NASA ER‐2 instrument suite. Between 30 and 40 degrees of freedom of signal are associated with the measurements, which is insufficient to provide full microphysics profiling. The measurements have the largest impact on the retrieval of ice particle sizes, followed by ice water contents. Ice densities are mainly driven by a priori assumptions, though low relative errors in ice densities suggest that in extensive regions of the convective system, only particles with densities larger than 0.4 g/cm^3^ are compatible with the observations. This is in agreement with reports of large hail on the ground and with hydrometeor classification derived from ground‐based polarimetric radars observations. This work confirms that multiple scattering generated by large ice hydrometeors in deep convection is relevant for airborne radar systems already at Ku band. A fortiori, multiple scattering will play a pivotal role in such conditions also for Ku band spaceborne radars (e.g., the GPM Dual Precipitation Radar).

## Key Points

- Novel technique to retrieve microphysics of graupel and hail in severe convection via the combination of microwave instruments
- Observations compatible with high‐density ice in agreement with ground reports and with polarimetric radar hydrometeor classification
- Multiple scattering generated by large ice hydrometeors in deep convection is relevant for airborne radar systems already at Ku band

## 1 Introduction

Deep convection plays a key role on the Earth system by shaping the weather and climate and by affecting the hydrological cycle and the composition of the atmosphere. Despite its importance, there is a dearth of observations of convective processes. On the other hand modeling of convective clouds is becoming increasingly sophisticated: regional‐scale numerical weather prediction models are now run at convection‐resolving scales, i.e., at horizontal resolutions of the order of 1 km [e.g., *Lean et al.*, 2008]. In addition, they are now moving away from predefined classes of hydrometeors (e.g., hail or graupel with prescribed density) toward explicitly predicting a continuum of hydrometeors properties like density and shapes [*Morrison and Milbrandt*, 2015; *Morrison et al.*, 2015].

In deep convective systems the strong coupling between microphysics and dynamics via latent heat release and condensate loading makes the representation of microphysics crucial for properly modeling the storm structure and its resultant precipitation. This has been highlighted by the fact that bulk and spectral‐bin model simulations are very sensitive to some settings like the ice partitioning into classes or the thresholds for conversion between ice species and parameter (e.g., fall speeds and particle densities). For instance, *Morrison and Milbrandt* [2011], *Adams‐Selin et al.* [2013], and *VanWeverberg* [2013] showed that different approaches in treating graupel and hail can lead to substantial differences in simulation outputs.

The new paradigm in cloud modeling calls for innovative observations which are capable of quantitatively characterizing hydrometeor properties like size, density, and equivalent water contents with a rigorous error assessment. Because of the safety concerns associated with aircraft penetrating convective cores, airborne remote sensing observations remain irreplaceable assets for understanding the vertical structure of dynamics and microphysics of deep convection. Previous studies have focused on examining the relation between the vertical structure of reflectivities and the radiometric response [*Heymsfield et al.*, 1996], on characterizing the strength of updrafts in different convective categories (tropical cyclone, oceanic, land, and sea breeze) [*Heymsfield et al.*, 2010], and on understanding the airborne Ku‐Ka dual‐frequency radar characteristics in hail‐bearing convective cells and their implication for ongoing spaceborne radar missions [*Heymsfield et al.*, 2013; *Battaglia et al.*, 2014].

Combined radar‐radiometer algorithms have shown potential for estimating hydrometeor profiles from airborne platforms [e.g., *Skofronick‐Jackson et al.*, 2003; *Jiang and Zipser*, 2006] and from spaceborne suites like those on board the TRMM and GPM satellite [*Haddad et al.*, 1997; *Grecu et al.*, 2004, 2009]. The rationale underpinning all such retrievals is that, differently from infrared and visible, microwave radiation can penetrate deep convective systems and profile the interior of a precipitating system. Scattering properties of millimeter‐ and centimeter‐size particles strongly depend on the wavelength of the impinging electromagnetic radiation. For instance, attenuation and scattering become more and more important when moving from X (8–12 GHz) to W (75–110 GHz) band [*Ulaby et al.*, 1986; *Lhermitte*, 1990; *Kollias et al.*, 2007], while radiation scattered by hydrometeors becomes increasingly scattered in the forward direction [*Battaglia et al.*, 2010]. Radars detect the range‐resolved backscattering of particles and are affected by the two‐way attenuation between radar transmitter/receiver and the target. As a result, the received power at different frequencies differs because of differences in the backscattering mechanism of the targets (the so called “non‐Rayleigh effect”) [*Bohren and Huffman*, 1983] and in the attenuation properties of the medium through which the electromagnetic wave has propagated. Because of the complexity in disentangling the two effects, retrieval algorithms typically have been developed for regimes where one effect is predominant. For instance, *Hogan et al.* [2005] and *Ellis and Vivekanandan* [2011] have proposed dual‐wavelength differential attenuation methods for the estimation of the cloud, drizzle, or light rain liquid water contents, while *Matrosov* [1998], *Wang et al.* [2005], and *Liao and Meneghini* [2011] have exploited non‐Rayleigh effects for sizing and for hydrometeor classification in snow and ice clouds. Only in the presence of additional information (high quality Doppler spectra) has a full disentangling between non‐Rayleigh and attenuation effects been proved possible [*Tridon et al.*, 2013] by following a technique analogous to the high spectral resolution lidar technique.

In deep convection not only large‐sized particles like hailstones are producing ubiquitous non‐Rayleigh effects but also attenuation at all frequencies currently deployed in airborne systems (i.e., at and above X band). In addition, when attenuation is caused by highly scattering particles like hailstones, multiple scattering (*M* *S*, a comprehensive review is provided by *Battaglia et al.* [2010]) effects can partially compensate for attenuation and reduce the radar ranging capabilities. When observing storms bearing high‐density frozen hydrometeors such a phenomenon has been proven relevant not only for spaceborne radars [*Battaglia et al.*, 2016, and references therein] but also for airborne radar systems [*Heymsfield et al.*, 2013; *Battaglia et al.*, 2014]. Thus, the problem of reconstructing the state of the atmosphere in such conditions is extremely challenging.

Additional vertically integrated information about the hydrometeor structure is therefore highly desirable to further constrain and stabilize the inversion problem [*Löhnert et al.*, 2001; *L'Ecuyer and Stephens*, 2002]. Examples of these are provided by radar path‐integrated attenuations (*P* *I* *A*), typically derived by the surface reference technique and by radiometer brightness temperatures (*B* *T*s). Note that the availability of multifrequency radiometric channels allows probing into the different hydrometeor layers of precipitating systems, though the upwelling BTs remain a complex function of the absorption/emission, scattering, and thermodynamic properties of the hydrometeors in the observed column and of the temperature and emissivity of the surface underneath. Not only do precipitation‐sized ice particles reduce the measured BT above a relatively warm background at higher frequencies (37 GHz and above) [e.g., *Spencer et al.*, 1989], but they can also affect the lower frequencies [*Mugnai et al.*, 1993].

The joint NASA Integrated Precipitation and Hydrology Experiment (IPHEx) and Radar Definition Experiment 2014 (RADEx'14) field campaign (details at http://pmm.nasa.gov/iphex) offers unprecedented observations of deep convective cores via four radar and radiometer channels with frequencies ranging from X to W band. This is an ideal test bed for better understanding the potential of multiwavelength suite of microwave active and passive observations in deep convection. By disentangling the different contributions of attenuation, non‐Rayleigh, and MS effects, this work aims at assessing the penetration capabilities of the different frequencies, the limitation in retrieving high rain rates and in constraining the density/characteristic size and mass of the ice phase, and the importance of integral constraints like brightness temperatures or PIAs in better defining the solution space.

The paper is organized as follows: Section 2 presents the IPHEx field campaign, a general overview for the case study, the airborne observations, and the measurement quality control procedure. Section 3 discusses the retrieval framework. Section 4 illustrates and discusses the retrieval results obtained for the case study. Finally, section 5 draws the major conclusions and recommendations for future research.

## 2 The IPHEx Field Campaign

The IPHEx/RADEx14 joint field campaign [*Barros et al.*, 2014] was conducted in the Eastern U.S. from 1 May to middle of June 2014. Among other goals, it aimed at assessing and improving the accuracy of existing precipitation algorithms for spaceborne sensors—including those on the GPM core observatory [*Hou et al.*, 2014]—and at developing new algorithms for novel measurement concepts, like the radar for the Aerosol‐Cloud‐Ecosystem (ACE) mission concept [*Tanelli et al.*, 2010].

In addition to a number of instruments that measure rain at the ground, the NASA ER‐2 plane was flying at an altitude of 20 km equipped with a suite of sensors:

1. Two radiometers: the Conical Scanning Millimeter‐wave Imaging Radiometer (CoSMIR) with channels in the high‐frequency band (85–183 GHz)—which unfortunately was not working during the flight here analyzed—and the Advanced Microwave Precipitation Radiometer (AMPR) with channels in the frequency band between 10 and 85 GHz [*Lang and Roberts*, 2015] (specifics provided in Table 1);
2. Three different radar systems: an upgraded Cloud Radar System (CRS) [*Li et al.*, 2004], the High‐Altitude Wind and Rain Profiler (HIWRAP) [*Li et al.*, 2016], and a new scanning ER‐2 X band Radar (EXRAD). All radars have Doppler capabilities and were operated in the same configuration, looking at the nadir and sampling data every 50 m along track. Specifics of the radars are listed in Table 2. Note that the CRS has the narrowest beam width and the highest sensitivity, which makes it an ideal instrument for profiling the upper part of convective systems. The HIWRAP works at frequencies of 13.9 GHz and 35.3 GHz, nearly identical to the GPM Core Observatory Dual‐Frequency Precipitation Radar. Its vertical resolution of 250 m matches the GPM configuration, while it is oversampled by a factor of 6.6 in order to improve surface measurements. The EXRAD complements the other radars by gathering data at 9.6 GHz frequency, better suited than the other ER‐2 radar wavelengths for hail and heavy rain detection.

**Table 1:** AMPR Performance Characteristics^a^

Frequency (GHz)	10.7	19.35	37.1	85.5
Bandwidth (MHz)	100	240	900	1400
Integration time (ms)	50	50	50	50
Beam width (deg)	8	8	4.2	1.8
Footprint at the ground (m)	2780	2780	1480	640

a A flying altitude of 20 km is assumed.

**Table 2:** Specifics of the Radar Systems Available on the ER‐2 for the IPHEx Flight^a^

Frequency Band (Radar)	X (EXRAD)	Ku (ER‐2 HIWRAP)	Ka (ER‐2 HIWRAP)	W (CRS)
Frequency (GHz)	9.6	13.9	35.3	94.0
Beam width (deg)	3	2.9	1.2	0.4
Vertical resolution (m)	84	255	255	100
Vertical sampling (m)	18.4	37.5	37.5	37.5
Sensitivity at 10 km (dBZ)	−7	−1	−19	−25
Footprint at the ground (m)	1047	1012	420	140

a A flying altitude of 20 km is assumed.

### 2.1 Case Study

**Figure 1:**
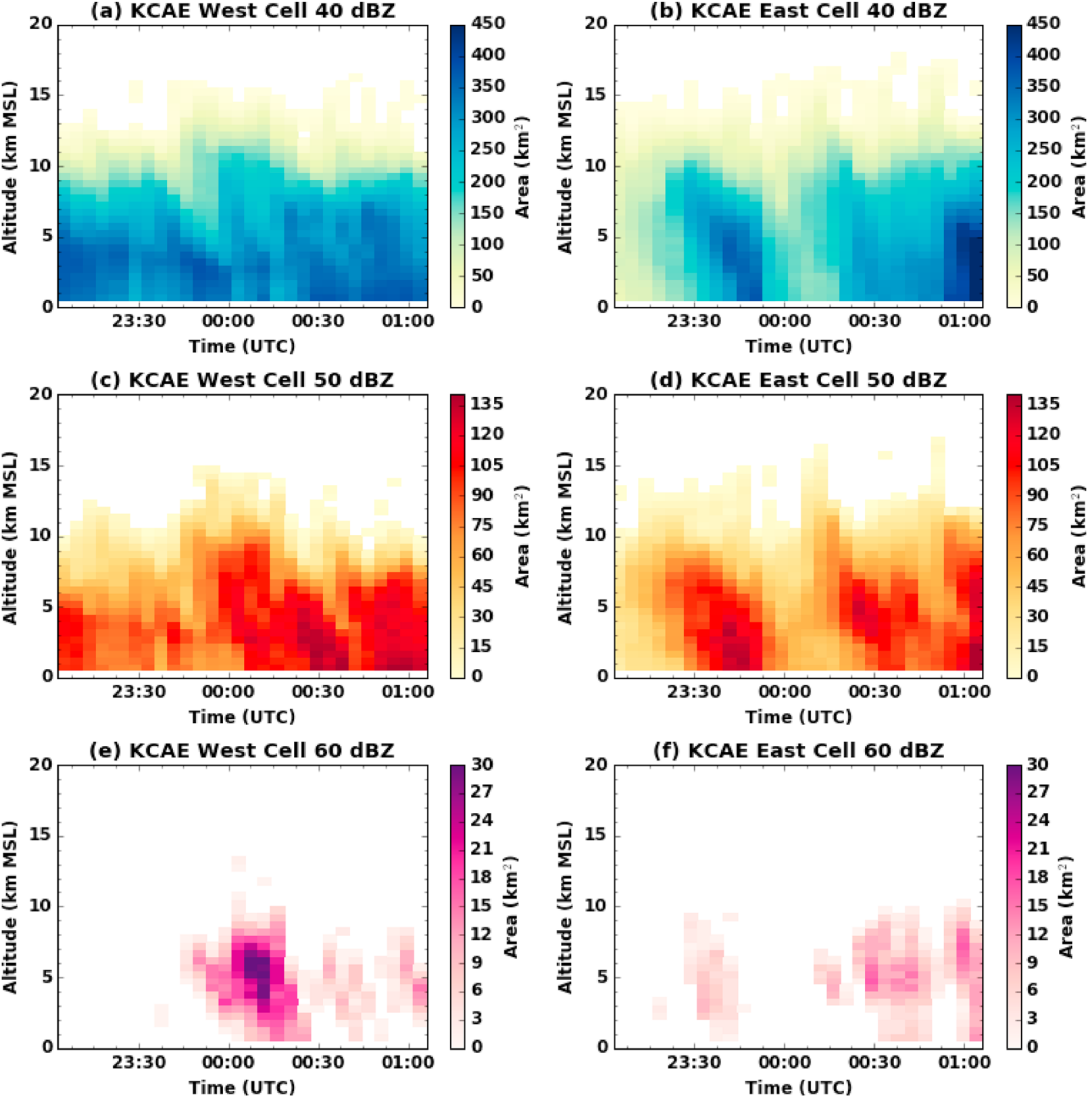
Time history of the area covered by reflectivities above (a, b) 40 dBZ, (c, d) 50 dBZ, and (e, f) 60 dBZ as a function of height for the (a, c, and e) western cell and (b, d, and f) eastern cell overflown by ER‐2. Data are from gridded KCAE S band (3 GHz, 10 cm wavelength) radar volumes.

On 23 May 2014, a weak backdoor cold front pushed through North Carolina. During the early morning hours, a weakening line of showers and thunderstorms moved into the North Carolina mountains from East Tennessee. Localized damaging winds were observed along the leading edge, even after the line had begun to dissipate as it approached the Blue Ridge Mountains. During the afternoon and evening, strong‐to‐severe convection triggered out ahead of this front thanks to some modest instability and vertical wind shear. Geostationary imagery shows the triggering of a convective area between 18:15 UTC and 18:30 UTC that by 24:00 UTC resulted in a well developed anvil (approximately 150 km in diameter) and with two strong convective cells and a half dozen of weaker ones (not shown). The two main cells, hereinafter referred to as the east cell and west cell, resulted in many reports of hail up to 5 cm diameter from the NOAA Storm Prediction center. The KCAE WSR‐88D S band (3 GHz, 10 cm wavelength) located in Columbia, South Carolina, acquired excellent data for these two cells at ranges between 10 and 50 km. Figure 1 shows the areal evolution of the high reflectivity portions of the two cells. This was calculated by interpolating the reflectivity data from each radar volume to a Cartesian grid, calculating areas above different reflectivity thresholds at each height level, and then manually tracking the cells' evolution from one volume to the next. In the period between 23:00 UTC and 01:00 UTC, the west cell had one short impulse with large hail (as inferable from the 60 dBZ area evolution, Figure 1e), and several cycles of weakening and strengthening that resulted in areas of several tens of square kilometers to exceed 50 dBZ above 10 km altitude. The east cell did not experience a similar extreme impulse, but it did nevertheless produce very high reflectivity signatures in the upper troposphere.

**Figure 2:**
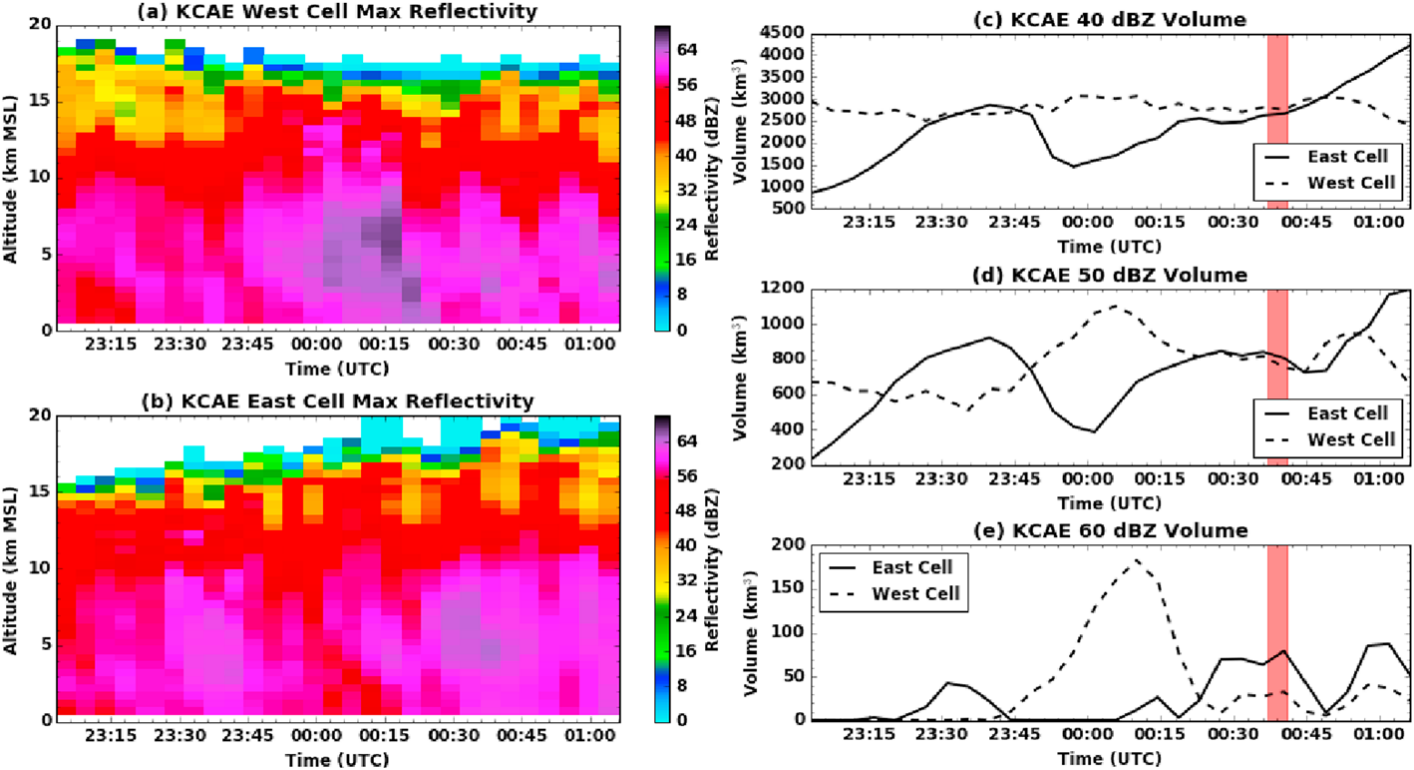
(a) Time history of maximum reflectivity as a function of height for the western cell. (b) Same as Figure 2a but for the eastern cell. (c) Total volume of 40 dBZ or greater reflectivity for the two cells. The 00:37–00:41 UTC overflight time is highlighted in red. (d) Same as Figure 2c but for 50 dBZ. (e) Same as Figure 2c but for 60 dBZ. Data are from gridded KCAE S band (3 GHz, 10 cm wavelength) radar volumes.

The time history of maximum reflectivity and of total storm volume above three different reflectivity thresholds is shown in Figure 2. The most notable results are that the west cell had a major hail episode well prior to the 00:40 UTC overflight, although it stabilized (the 40 dBZ volume (Figure 1b) changed little between 23:00 and 01:00 UTC) and even has begun strengthening again at the time of the overflight (see the reappearance of reflectivities above 60 dBZ, Figure 1e). The east cell undergoes a split prior to 00:00 UTC which is highlighted by the sudden decrease in intensity and storm volume around that time (blue lines in right panels). After 00:00 UTC the east cell starts recovering from the split and is intensifying by the time of the overflight. After 01:10 UTC, the two cells of interest began a merger process with each other.

**Figure 3:**
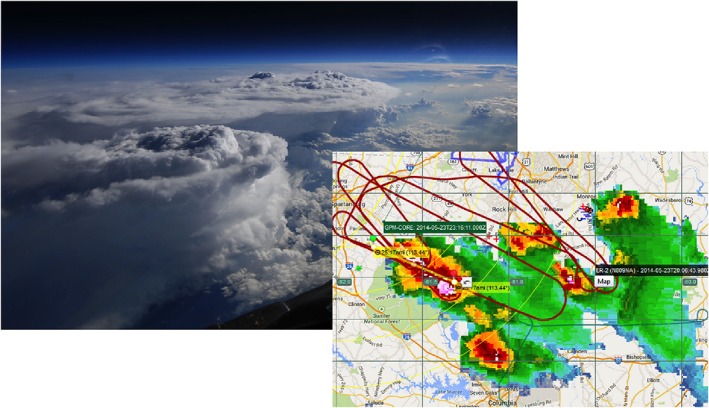
Aircraft track as seen in the Operations Control center monitors, as well as a photo of the storm from the ER‐2 cockpit (photo credit and courtesy of Donald Stuart “Stu” Broce, Armstrong Flight Research Center). The photo was taken looking to the southeast, so the plane was likely on a left turn and pointing somewhere around SW at the time of picture.

**Figure 4:**
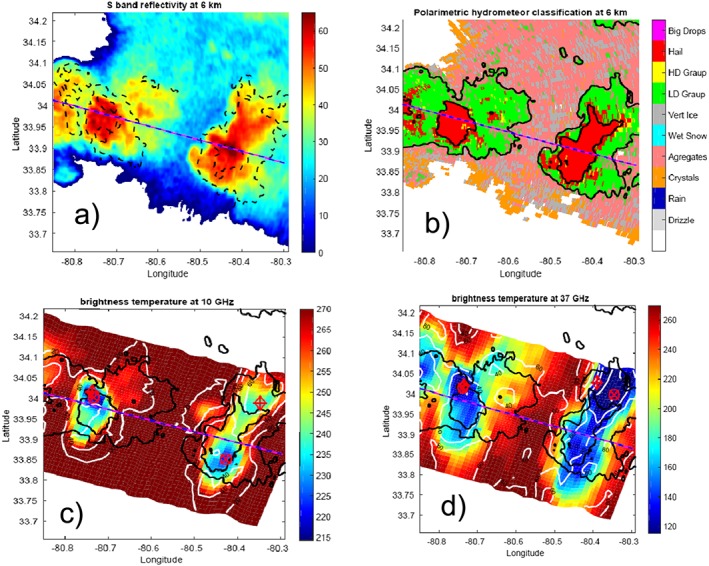
Horizontal cross section of (a) reflectivity and of (b) hydrometeor classification at 6 km above ground level from KCAE radar. The dashed red‐blue line corresponds to the ER‐2 flight tracks during 00:37–00:41 UTC, while the dashed (continuous) black lines in Figure 4a (Figure 4b) correspond to the contour levels for KCAE radar reflectivity at 10 km (6 km) altitude. The (c) 10 and (d) 37 GHz BT as measured by AMPR. The black lines correspond to the 30 and 50 dBZ contour levels of the reflectivities at 6 km, while the white contour lines are the BT difference between the 10 and 19 GHz channels (Figure 4c) and between the 37 and 85.5 GHz channels (Figure 4d). Circles (diamonds) with crosses correspond to the minimum of 10 (19) and 37 (85.5 GHz) AMPR channels, respectively.

The ER‐2 performed many overpasses of these storms from 23 May to early 24 May as they propagated south‐southeastward from North Carolina to South Carolina. Figure 3 shows the aircraft track as seen in the Operations Control Center monitors, as well as a photo of the storm from the ER‐2 cockpit, featuring the two largest cells of the storm. This work focuses on the ER‐2 data acquired between 00:37 and 00:41 UTC. As highlighted by its ground track shown in Figure 4, the ER‐2 is flying toward the E/S‐E direction and is passing over the core of the east and west cells, located at latitude‐longitude coordinates of about (34°, −80.75°) and (33.9°, −80.45°). The 51 km long track is passing over some forested areas at longitude −80.8° (on the left) and then some agricultural patches and urban areas (suburbs of Sumter, North Carolina) at longitude −80.3° (on the right). The horizontal structure of the system is clearly illustrated by the 6 km height cut observed by the NEXRAD S band located at the KCAE Radar Station (33.949°N, 81.119°W). The volume scan closest‐in‐time to the ER‐2 observations was performed by the radar between 00:36:21 UTC and 00:40:15 UTC. It clearly highlights two intense convective cores (red/dark red areas): the west cell is smaller in size and has smaller maximum reflectivities (71 dBZ reached at 3 km) than the east cell (74 dBZ reached at 3 km), but it is equally, if not more, vertically developed. In both cells the reflectivities are still above 45 dBZ at 10 km altitude as shown by the black dotted contour lines.

The brightness temperatures (Figures 4c and 4d) show very cold depression in correspondence to the two cores, with values as low as 214 K (223 K), 164 K (163 K), 115 K (95 K), and 91 K (56 K) for the 10, 19, 37, and 85.5 GHz channels, respectively, for the west (east) core. According to *Leppert and Cecil* [2015, Figure 13] these values are only compatible with hail or high‐density graupel. Similarly, *Cecil* [2009] showed that such low BT values, especially at 37 and 19 GHz, are clear signatures of millimeter‐sized particles in the upper portions of clouds. Interestingly, while the maxima of BT depressions are almost overlapping and located north of the ground track for the west cell, this is not true for the east cell where the absolute minimum is found south of the ER‐2 track for the 10 GHz channel (circle with cross in Figure 4c), while it is at the northern edge of the swath for the other three channels.

### 2.2 Nadir Curtain

**Figure 5:**
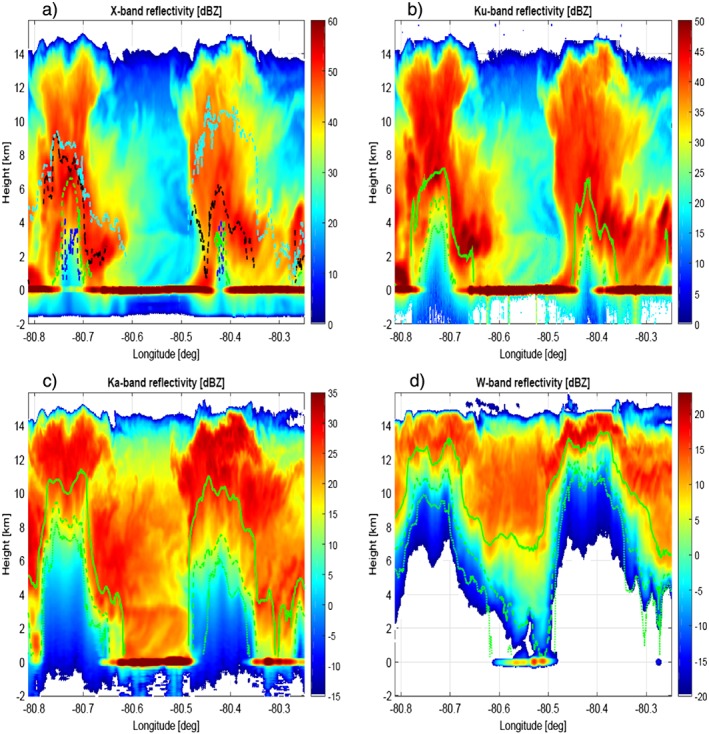
Time‐height reflectivity profiles for the IPHEx overpass shown in Figure 4 for the four radar channels. (a) The different lines (blue, green, black, and cyan) correspond to the level below which the multiple scattering contribution become predominant at X, Ku, Ka, and W band, respectively (see text in section 4 for details). (b–d) The continuous, dashed, and dotted green lines correspond to the levels at which the top‐down optical thickness exceeds 1, 3, and 5 (see discussion in section 4).

**Figure 6:**
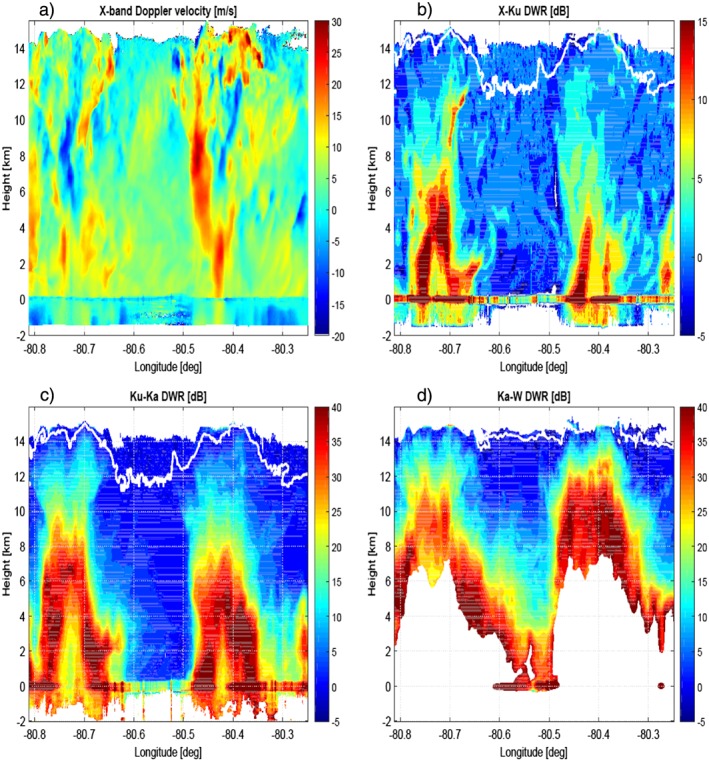
(a) X band time‐height mean Doppler velocity profiles and the three DWRs corresponding to (b) X‐Ku, (c) Ku‐Ka, and (d) Ka‐W. Doppler velocities are negative when toward the radar and therefore for upward motions. The calibrating Rayleigh regions for DWRs are delimited by the areas above the white lines.

**Figure 7:**
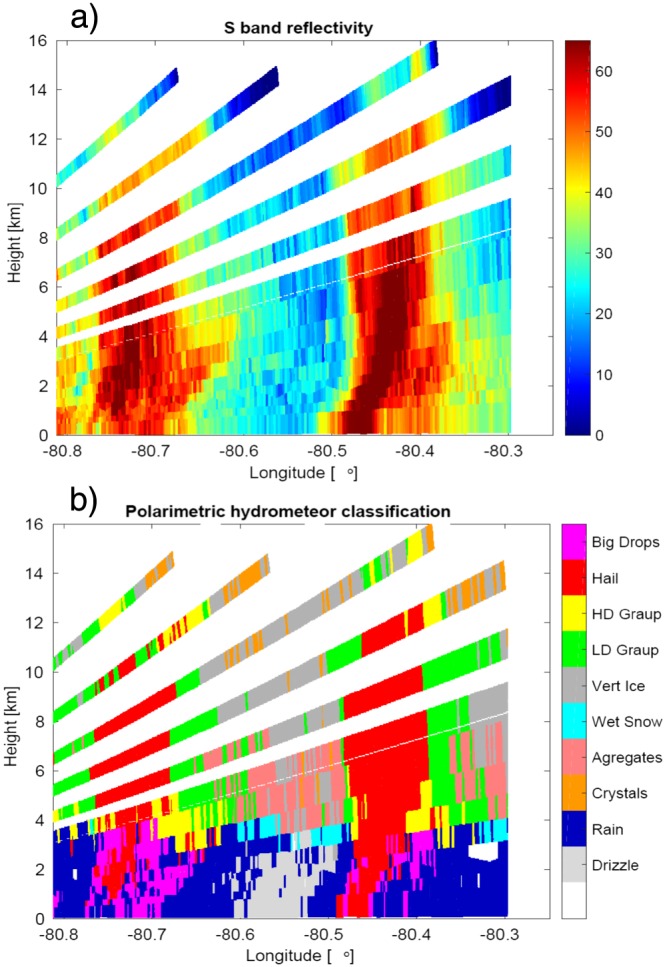
Vertical curtain of (a) S band reflectivities and (b) hydrometeor classification reconstructed from the ground‐based KCAE radar along the ER‐2 flight.

The ER‐2 radars were configured to look only at nadir. The nadir curtain radar observations are depicted in Figures 5 and 6, while the reconstructed S band reflectivity and the hydrometeor classification derived from the ground‐based polarimetric observables are shown in Figure 7. The ground‐based data suggest the presence of two hail shafts, one at about LON −80.77° and the other just before LON −80.47°. This is supported by the X and Ku band profiles (Figures 5a and 5b) that are not as strongly affected by attenuation as the regions at the center of the convective cores and by the very high downward X band Doppler velocities (Figure 6a). The ground‐based radar hydrometeor classification (via the algorithm used by *Tessendorf et al.* [2005], with an additional Big Drops category following S band updates to *Dolan et al.* [2013]; Figure 7b) suggests that the two hail shafts were embedded in two heavy rain pockets. This is confirmed by the airborne observations, with an attenuation signal clearly visible at X band already below 4(3) km for the west (east) cell. Similarly, the Ku channel is affected by strong attenuation in correspondence to the center of the two cells below 6/5 km. The higher‐frequency (Ka and W) radars on the other hand (Figures 5c and 5d) are suffering from strong attenuation already in the upper levels. For instance, for the W band, strong attenuation is already evident at 12 km height within the convective cores, which rapidly drives the radar signal below noise already at 7 km. At W band the attenuation is so strong within all the scene that only in the region between LON −80.61° and LON −80.48° was a return signal from the surface above the radar noise level. The convective nature of the whole scene is confirmed by the X band Doppler velocities (Figure 6a) with downward and upward Doppler velocities exceeding 30 and 20 m/s, respectively.

### 2.3 Data Resampling and Cross Calibration

All radars that were flying on ER‐2 plane had different antenna beam widths (see Table 2), and they sampled data at different ranges and at different time stamps. Therefore, data were regridded by convolving measured reflectivities with appropriate averaging functions. Measurements made with narrower beam widths do not provide full coverage of the footprints of channels with wider beam widths; this effect can be partially mitigated by adopting a two‐dimensional along‐track approach, which accounts for the Gaussian form of the two‐way antenna gain functions:

(1) $$G_{\theta_{\text{3dB}}}^{2}(\theta )=\frac{\sqrt{8\ln2}}{\sqrt{\pi \theta_{\text{3dB}}^{2}}}\exp\left(-8\ln(2){\left(\frac{\theta }{\theta_{\text{3dB}}}\right)}^{2}\right),$$

where *θ*_3dB_ is the 3 dB antenna beam width (reported in the third row of Table 2 for the ER‐2 radars). The convolution of two Gaussian probability density functions is a Gaussian probability density function. When applied to the gain function of the Ku antenna, this property can be expressed as

(2) $$G_{\theta_{\text{Ku}}}^{2}\propto G_{\theta_{W}}^{2}*G_{\gamma_{1}}^{2},\;G_{\theta_{\text{Ku}}}^{2}\propto G_{\theta_{\text{Ka}}}^{2}*G_{\gamma_{2}}^{2},$$

where $\gamma_{1}=\sqrt{\theta_{\text{Ku}}^{2}-\theta_{W}^{2}}$, $\gamma_{2}=\sqrt{\theta_{\text{Ku}}^{2}-\theta_{\text{Ka}}^{2}}$. Therefore, in order to regrid the Ka and W band data to the Ku beam width W and Ka band reflectivities are convolved in linear units with $G_{\gamma_{1}}^{2}$ and $G_{\gamma_{2}}^{2}$, respectively. X band measurements were not processed because of insignificant differences in antenna patterns between X and Ku antennas.

In order to overcome the different vertical resolutions, the highest‐resolution data at X and W band (see fourth row of Table 2) were convolved with a characteristic top‐hat function of a range of (−255/2,255/2) m. Then resulting reflectivities are linearly interpolated on the Ku 37.5 m sampling grid.

**Figure 8:**
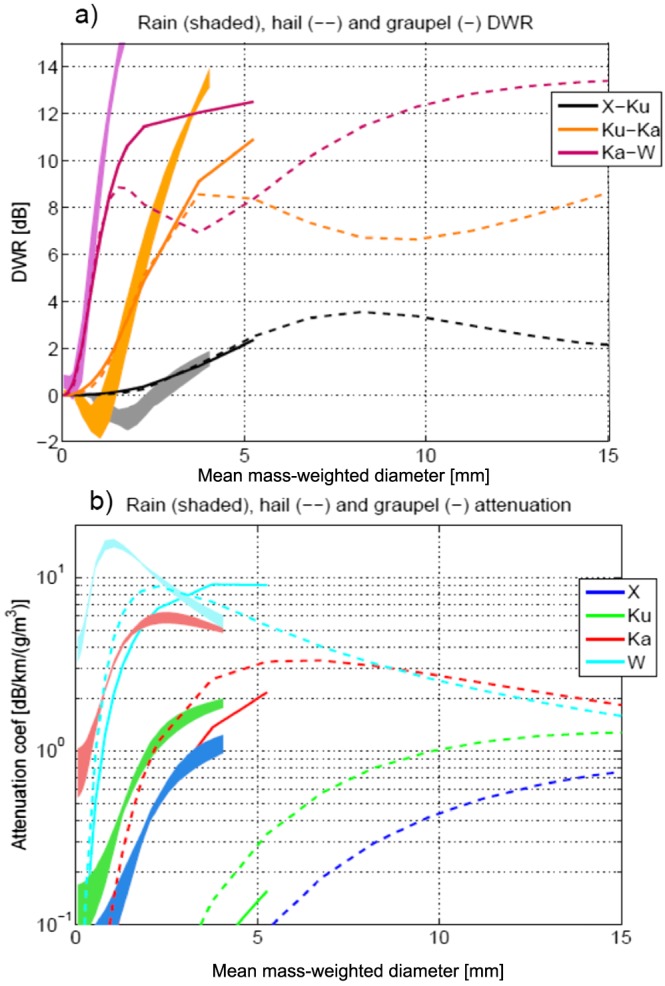
DWR for the three contiguous pair of the ER‐2 radars and attenuation coefficients as a function of the PSD mass‐weighted mean diameter for the four ER‐2 radar frequencies. Continuous and dashed lines correspond to exponentially distributed graupel (density 0.4 g/cm^3^) and hail (density 0.9 g/cm^3^) PSD; shaded envelopes encompass the variability for *Γ*‐distributed rain with *μ* between 0 and 3 and temperatures in the range between 0° and 30°C. *D*_*m*_ are stopped at 4 mm and 5 mm for rain and graupel, respectively.

Radars were not fully cross calibrated during the flight. In order to resolve this issue a simple “constant shift” approach was used for the entire scene under consideration similarly to *Tanelli et al.* [2006]. The Ku channel is considered as the reference because it was externally calibrated during the flight using the ocean‐surface return, with an estimated accuracy of ±1 dB. To calibrate the Ka channel regions at great heights in the atmosphere are selected, where both signals achieved good signal to noise ratio (e.g., above −1 dBZ for Ku and above −19 dBZ for Ka). Then this domain has been further restricted to the region of relatively small reflectivity values where Rayleigh scattering is expected for both channels, e.g., *Z*_Ku_<14 dBZ for the Ku‐Ka pair. The most frequent dual‐wavelength ratio (*D* *W* *R*) between the Ka and Ku reflectivity signal in this domain was treated as an offset and was added to the Ka channel measurements. The same approach was used for cross calibrating the X‐Ku and Ka‐W reflectivity pairs with the Rayleigh criterion specified by the conditions: *Z*_Ku_<14 dBZ and *Z*_Ka_<−4 dBZ, respectively. The three cross‐calibrated DWRs corresponding to the three contiguous frequency channels are plotted in Figures 6b–6d with the calibrating Rayleigh regions delimited by the areas above the white lines. The distribution of DWRs within the calibration domains is Gaussian, centered around zero with mean standard deviations of 1.51, 1.49, and 4.3 dB for the X‐Ku, Ku‐Ka, and Ka‐W, respectively. The W band calibration appears to be the most troublesome one for the paucity of points available. DWRs generally look sensible with values increasing when moving downward inside the convective cores as a result of combined non‐Rayleigh and attenuation effects [e.g., see *Battaglia et al.*, 2014; *Tridon et al.*, 2013]. However, in correspondence to the center of the cells (especially for the west one), at heights close to the surface, the reverse is observed for all three DWRs. This reflects the appearance of a DWR “knee,” a characteristic signature that MS contributions have become so dominant in the highest frequency that they substantially compensate for the attenuation and the decrease rate of reflectivities toward the ground is larger at the lowest frequency [*Battaglia et al.*, 2014, 2015]. Importantly, the pronounced “knee” at about LON −80.73° in the X‐Ku DWR (Figure 6b) provides strong indication that MS is significantly affecting the Ku signal in deep convective cores, as previously noted by *Battaglia et al.* [2016]. Outside of the two convective cores the DWR of the two lowest pairs is not dominated anymore by attenuation effects; for instance, the positive Ku‐Ka DWR values (Figure 6c) corresponding to the protuberance between 8 and 10 km height around LON −80.34° are clearly produced by non‐Rayleigh effects. Similarly for some slightly negative values of X‐Ku DWR which occur in the rain layer with longitudes between −80.62° and −80.48°. This is expected from theoretical computations as shown in Figure 8a. Anomalous negative and highly positive values of DWRs (with the latter not being caused by differential attenuation) especially for the pair X‐Ku are, however, present in the scene, particularly in regions characterized by strong spatial variability (e.g., edges of clouds and precipitating cells like at 9–12 km height on the right flank of the west cell at around LON −80.68° and at 6–8 km height on the left flank of the east cell at LON −80.48°). This is likely due to the antenna and range resolution mismatches for the different radar wavelengths.

### 2.4 PIA Estimation

**Figure 9:**
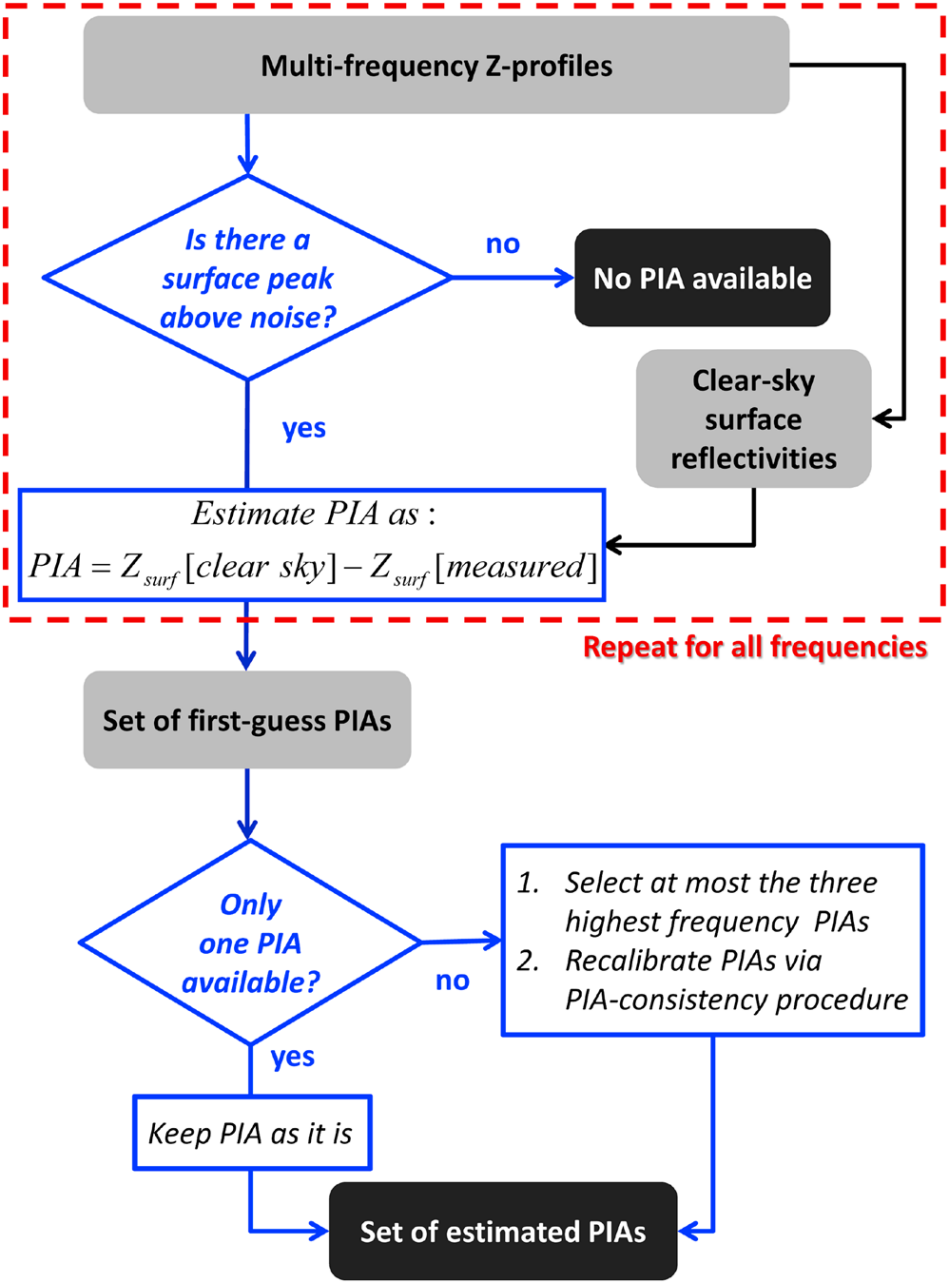
Flow chart for the PIA estimation.

PIA estimates based on the surface reference technique are particularly challenging over land. The surface return in fact can be affected by very reflective objects and by a wet ground after a storm passes over. A multiwavelength approach can help in this respect as well. The path‐integrated attenuations are estimated following a two‐step procedure described in the flow chart depicted in Figure 9. First, surface reflectivity peaks above the noise level are searched for in the reflectivity profiles at all radar frequencies. Only those frequencies whose signal from surface ranges is significantly above the noise threshold and does not exhibit MS behavior (i.e., absence of a peak) are considered further for providing useful PIAs. For such frequencies PIAs are simply estimated by using as a reference an average value of surface reflectivity in clear sky conditions during the same flight.

**Figure 10:**
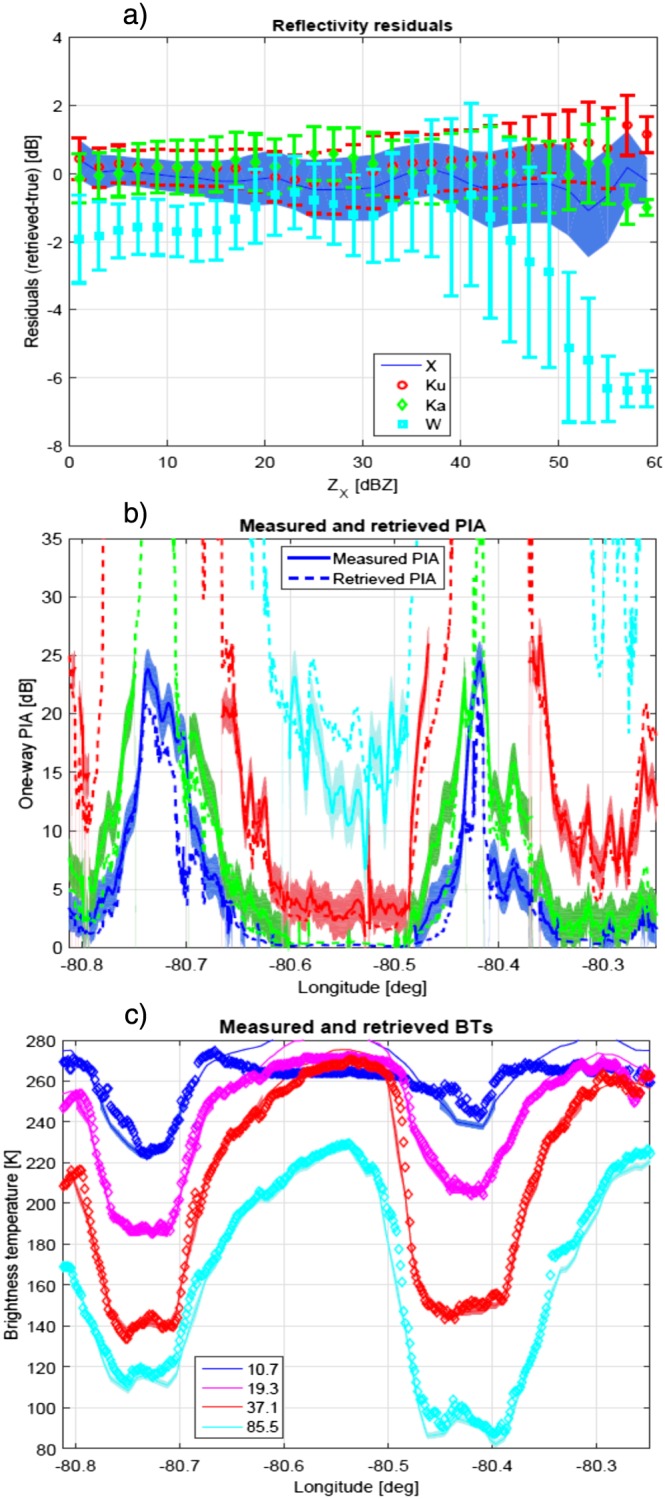
(a) Reflectivity residuals as a function of the X band reflectivity. (b) Measured and retrieved PIAs. Results have been truncated at 35 dB because observed PIAs do not typically exceeds 26 dB. (c) Measured (continuous lines with errors shaded) and retrieved (diamonds) BTs.

Second, the set of PIAs derived from the first step is then passed through a quality control procedure. If more than one PIA is available (but, because of the noisiness of the one corresponding to the lowest frequency we consider at most three of them, corresponding to the three highest frequencies), PIAs are recalibrated according to a consistency procedure among the different channels. Each PIA is perturbed by the same amount, *δ*PIA; the procedure is repeated until the ratios between all contiguous frequency PIA pairs fall within fixed ranges preliminarily determined by scattering computations and PSD observations. For the Ku‐X, Ka‐Ku, and W‐Ka these are [1.75,2.4], [3,11], and [3,8], respectively. The PIAs that fulfill such requirements and that correspond to the minimum absolute change of *δ*PIA are selected as set of estimated PIAs. Finally, an 11‐bin running average is performed to smooth the PIA field. Estimates of PIAs and corresponding errors are plotted later on in Figure 10b (continuous lines and shadings).

## 3 Retrieval Algorithm

In order to get an insight into the microphysical structure of the two convective cells a microphysics retrieval algorithm has been developed. The methodology is based on an optimal estimation approach [*Rodgers*, 2000], which has the flexibility of integrating different sensors (here radar and radiometer) in a physically consistent way, i.e., by accounting for the accuracy and information content of each different measurement and with the advantage of providing a rigorous framework for computing retrieval errors. Optimal estimation techniques have been successfully applied in the area of cloud/precipitation remote sensing [*Delanoë and Hogan*, 2008; *Munchak and Kummerow*, 2011] though only few have applied it to multiwavelength radar and radiometer measurements [*Grecu et al.*, 2011; *Battaglia et al.*, 2015; *Mace et al.*, 2016].

The general inverse problem consists in retrieving an unknown state vector **x** (length *n*) from a measurement vector **y** (length *m*) which is characterized by a measurement error ***ε***, i.e., **y** = **F** **(** **x** **)** + ***ε***, where **F** represents a forward operator mapping state into measured variables. The retrieval problem is equivalent to minimizing the following cost function:

(3) $$\text{CF}=\underset{{\text{CF}}_{\text{meas}}}{\underset{︸}{{\left[y-F(x)\right]}^{T}S_{\varepsilon }^{-1}[y-F(x)]}}+\underset{{\text{CF}}_{\text{a‐priori}}}{\underset{︸}{{\left[x-x_{a}\right]}^{T}S_{a}^{-1}[x-x_{a}]}},$$

where **S**_*ε*_ is the measurement error covariance matrix and **x**_*a*_ is the a priori value of **x** and **S**_*a*_ the associated covariance matrix. Note the two contributions to the cost function coming from the measurements and from the a priori. A normalized cost function for measurements, ${\text{CF}}_{\text{meas}}^{n}$, obtained by dividing CF_meas_ by the length of the vector **y**, will be used in the following sections. The a priori term is used to partially constrain the problem and to prevent nonphysical values from being retrieved (e.g., ice densities exceeding 0.917 g/cm^3^).

The solution can be found by Newtonian iterations based on the Gauss‐Newton method [*Rodgers*, 2000]:

(4) $$x_{i+1}=x_{i}+{\hat{S}}_{i}\left[J_{i}^{T}S_{\varepsilon }^{-1}(y-{F(x}_{i}))-S_{a}^{-1}(x_{i}-x_{a})\right],$$

where **J**_*i*_ is the Jacobian of the forward operator evaluated at the *i* iteration of the state vector, **x**_*i*_, and

(5) $${\hat{S}}_{i}\equiv {\left(S_{a}^{-1}+J_{i}^{T}S_{\varepsilon }^{-1}J_{i}\right)}^{-1}$$

is the covariance matrix of the solution. The convergence criterion is based on checking when the covariance‐weighted square difference between successive estimates becomes much less than the number of retrieved parameters:

(6) $$d_{i}^{2}={\left(x_{i}-x_{i+1}\right)}^{T}{\hat{S}}_{i}^{-1}(x_{i}-x_{i+1})\ll n.$$

### 3.1 Vectors of Unknowns

For the specific problem in consideration we selected as unknowns the profiles of particle size distributions (PSDs) of rain and frozen hydrometeors, profiles of cloud liquid water and ice particle density (*ρ*_ice_). The PSDs are assumed to be exponential for the ice particles and *Γ* functions with *μ* = 3 for rain [*Liao et al.*, 2014]. With these choices PSDs are only functions of two parameters. We describe them in terms of the equivalent water content (*W* *C*) and the mass‐weighted mean diameter (*D*_*m*_), which have a simple physical interpretation. Accordingly, *the state vector* is

(7) $$y=\left(\begin{array}{c} \left.\begin{array}{c} D_{m}^{i}[1]\\ \vdots \\ D_{m}^{i}[N_{i}]\\ \underset{10}{\log}\text{IWC}[1]\\ \vdots \\ \underset{10}{\log}\text{IWC}[N_{i}]\\ \rho^{i}[1]\\ \vdots \\ \rho^{i}[N_{i}] \end{array}\right\}\text{ice}\;\\ \left.\begin{array}{c} D_{m}^{r}[1]\\ \vdots \\ D_{m}^{r}[N_{r}]\\ \underset{10}{\log}\text{RWC}[1]\\ \vdots \\ \underset{10}{\log}\text{RWC}[N_{r}] \end{array}\right\}\text{rain}\;\\ \left.\begin{array}{c} \underset{10}{\log}\text{CWC}[1]\\ \vdots \\ \underset{10}{\log}\text{CWC}[N_{c}] \end{array}\right\}\text{cloud} \end{array}\right),$$

where the logarithm of the WCs is taken in order to avoid the occurrence of nonphysical negative values. Note that ice, rain, and cloud are hydrometeors defined on a different number of levels (respectively *N*_*i*_, *N*_*r*_, and *N*_*c*_) as defined by the a priori (see discussion below).

### 3.2 Measurements

The *measurement vector* is a combination of radar reflectivities, PIAs, and brightness temperatures in the form

(8) $$y=\left(\begin{array}{c} \left.\begin{array}{c} Z_{m}^{X}[r_{1}]\\ \vdots \\ Z_{m}^{X}[r_{N_{X}}]\\ Z_{m}^{\text{Ku}}[r_{1}]\\ \vdots \\ Z_{m}^{\text{Ku}}[r_{N_{\text{Ku}}}]\\ Z_{m}^{\text{Ka}}[r_{1}]\\ \vdots \\ Z_{m}^{\text{Ka}}[r_{N_{\text{Ka}}}]\\ Z_{m}^{W}[r_{1}]\\ \vdots \\ Z_{m}^{W}[r_{N_{W}}]\\ {\text{PIA}}^{X}\\ {\text{PIA}}^{\text{Ku}}\\ {\text{PIA}}^{\text{Ka}}\\ {\text{PIA}}^{W} \end{array}\right\}\text{radar}\;\\ \left.\begin{array}{c} T_{B}[10.7\;\text{GHz}]\\ T_{B}[19.3\;\text{GHz}]\\ T_{B}[37.1\;\text{GHz}]\\ T_{B}[85.5\;\text{GHz}] \end{array}\right\}\text{radiometer} \end{array}\right),$$

where the measured reflectivities have been resampled at the same ranges *r*_1_,…,*r*_*S*_. Only reflectivities with good signal‐to‐noise ratio are considered, thus restricting the number of reflectivities to *N*_X_, *N*_Ku_, *N*_Ka_, and *N*_W_ for the four radar frequencies, respectively. Reflectivity errors are assumed to be 1 dB except for the W band channel and in correspondence to the ice‐to‐liquid transition layer where they are assumed to increase to 1.5 dB. For the W band channel the errors are increasing further to 2 dB at reflectivities below −20 dBZ. This is due to increasing modeling uncertainties in the MS‐contaminated tail.

PIAs have been estimated according to the procedure described in section 2.4. A 1.5 dB error is assumed for all PIAs. Brightness temperatures are considered only when lower than 250 K; otherwise, the uncertainties in surface emissivity introduce too much uncertainty in the brightness temperature modeling. Modeling errors on BTs are assigned to be 1.5 K below 240 K, linearly increasing up to 3 K at 250 K. In addition to these, interpolation errors are included due to the fact that brightness temperatures at the radar time stamps are linearly interpolated in time from the two closest radiometer time stamps of nadir observations. Such errors are assumed to be 20% of the absolute difference between the interpolated BT and the closest BTs used for the fitting. Errors on reflectivities at different range gates, PIAs and BTs are assumed to be independent so that the measurement error covariance matrix can be written in the block diagonal form:

(9) $$S_{\varepsilon }=\left[\begin{array}{ccc} S_{Z} & 0 & 0\\ 0 & S_{\text{PIA}} & 0\\ 0 & 0 & S_{T_{B}} \end{array}\right]$$

where *S*_*Z*_, *S*_PIA_, and $S_{T_{B}}$ are diagonal matrices, whose diagonal elements are determined by the previously discussed errors.

### 3.3 Forward Model

The code developed in *Hogan and Battaglia* [2008] is adopted as forward operator for computing the reflectivity profiles, the PIAs, and via the perturbation method the relevant Jacobians. The code accounts for MS effects, which can be relevant for millimeter‐wavelength airborne radar observations of convective cores as demonstrated in *Battaglia et al.* [2014]. The brightness temperatures are computed by an Eddington approximation code [*Kummerow*, 1993]. A planar‐stratified model is deemed acceptable in this work because of the small footprints of the instruments and because only high‐resolution nadir observations are used.

### 3.4 A Priori

Retrievals in convective cores are particularly challenging due to an increased range in the ice densities caused by the possible occurrence of graupel or hail, to the presence of occasionally large amounts of cloud liquid water, and to the large spatial variability of the PSD. Thus, we have decided to explore the space of possible solutions by adopting different a priori profiles based on different ice densities (ranging from 0.05 to 0.9 g/cm^3^), different characteristic mass‐weighted mean diameters compatible with the ice densities (e.g., graupel‐like particles having *D*_*m*_ not larger than 4 mm), and different liquid‐to‐solid transition heights. Rain (ice) is assumed to be present only below (above) a certain height, with a layer of coexistence between the ice and rain which is typically 1.5 km thick. Supercooled cloud liquid water is assumed to be present only above the freezing and below the −40°C level.

There is also an a priori which allows high‐density ice (hail) to be present down to the surface level. In this case no rain is present in the profile. This is to account for profiles where hail is the dominant species throughout the entire profile.

The a priori is driven by the low frequency X band radar observations that are not affected by attenuation and MS effects except for deep inside the two convective cores. The density is assumed to increase from the top of the cloud downward reaching the maximum at the top of the liquid‐to‐solid transition region. The increase of density is triggered by the increase in X band reflectivity. For instance, densities of 0.4 (0.9) g/cm^3^ are triggered only if X band reflectivities exceed 36 (42) dBZ, respectively. This is in line with ground‐based polarimetric radar hydrometeor classifications at this frequency [e.g., *Dolan and Rutledge*, 2009]. Similarly, *D*_*m*_ for the ice particles is increasing toward its maximum characteristic value at the reflectivity peak, and it is then decreased toward zero in the solid‐liquid precipitation coexistence region. The *I* *W* *C* is then computed by matching the X band reflectivities at each level. With the development of spectral microphysical modeling for hail‐bearing clouds [e.g., *Ryzhkov et al.*, 2013], it will be possible to construct these a priori based on microphysical consistent profiles. More work is certainly needed in this area.

### 3.5 Retrieval Errors

Convergence is generally found for different initial a priori assumptions but with different values for the cost function. We want to downselect only those solutions which are best fitting the measurements, i.e., which are minimizing CF_meas_ (see equation (3)). We consider as ensemble of solutions those whose CF_meas_ is within 25% from the minimum CF_meas_. If **X**_1_, …, **X**_*J*_ are these best *J* solutions, the final estimate is given by the mean of the ensemble:

(10) $$\bar{X}=\frac{1}{J}\overset{J}{\underset{i=1}{\sum }}X_{i}.$$

Note that if some solutions do not include some variables at certain levels, then they are treated as missing numbers and excluded from the averaging procedure. The estimate of the uncertainty on $\bar{X}$ is provided by summing quadratically the mean error of the different **X**_*i*_ profiles as derived by the optimal estimation procedure in the covariance matrix of the solution (equation (5)) and the standard deviation of the ensemble of solutions.

(11) $$\varepsilon_{\bar{X}}=\sqrt{{\left[{\bar{\varepsilon (X_{i})}}_{\left|i=1,\ldots J\right.}\right]}^{2}+{\left[\text{std}\left(X_{1},\ldots ,X_{J}\right)\right]}^{2}}.$$

The total uncertainty therefore accounts both for the spread of the attractors in the solution space and for the typical uncertainty in the retrieval within each single attractor propagated from the measurement and modeling errors into the observation space.

### 3.6 Example of Retrieval

**Figure 11:**
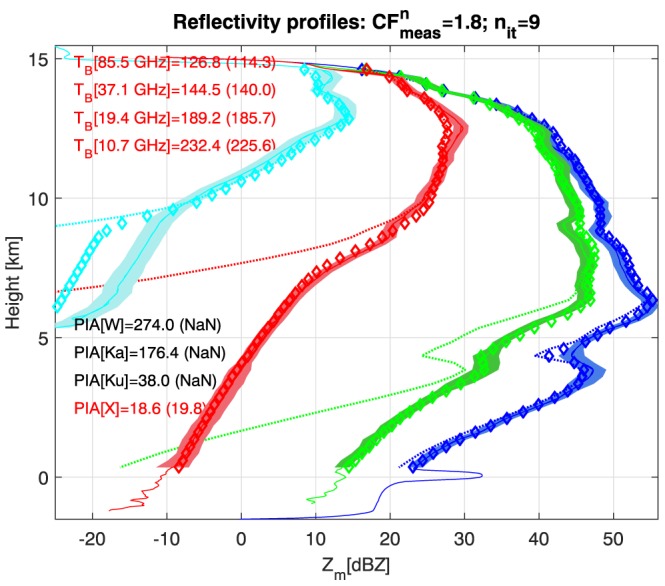
Retrieved (diamonds) and measured (continuous line with shading for the std) reflectivities for the profile corresponding to LON −80.7425°. Only the solution which is best fitting the measurements is shown. Dotted lines correspond to the single‐scattering retrieved reflectivities. Blue, green, red, and cyan lines correspond to X, Ku, Ka, and W band, respectively. The text on the left side of the panel shows retrieved (measured) BTs and PIAs. Only the measurements printed in red are used for constraining the retrieval.

The profile at LON −80.7425°, at the center of the west convective core, is used hereafter for showcasing the retrieval outputs. The measured reflectivity profiles (continuous lines in Figure 11) are characterized by a very high reflectivity region just above 6 km with reflectivities at X band exceeding 55 dBZ. All BTs (reported in parenthesis on the inset text in the top left corner) show significant depressions. Both features are clear symptoms of large dense ice particles aloft. Only the X band PIA is available. For Ku and Ka bands, though a signal well above noise is clearly detectable, no clear peak is visible at the surface range. Therefore, the criterion for proceeding to the second step of the PIA estimate (Figure 9) is not fulfilled. The disappearance of the surface returns both at Ku and at Ka but not at X (see continuous lines in correspondence to a height of 0 km in Figure 11) is a well‐known signature of strong MS effects, as already noticed in previous papers [*Battaglia and Simmer*, 2008; *Battaglia et al.*, 2014]. The X band PIA estimated value (19.8 dB) is quite remarkable and seems to suggest the presence of a strongly attenuating layer with heavy rain. This estimate of attenuation is also consistent with the strong decrease of reflectivity when moving from 6 km to the surface. Despite this very large PIA, however, only the W band channel is completely attenuated with the signal dropping below −25 dBZ at 5 km. Simple conversion of attenuation from one band to the other would predict complete attenuation of Ka and Ku channels as well, but this is not observed: in the lowest 5 km the slopes of the reflectivity profiles are indeed counter intuitively increasing with decreasing frequencies. Again, this anomalous behavior of the Ku and Ka band sloping compared to the X band is consistent with the presence of considerable MS enhancement in these two higher frequencies [*Matrosov et al.*, 2008; *Battaglia et al.*, 2014, 2015].

**Figure 12:**
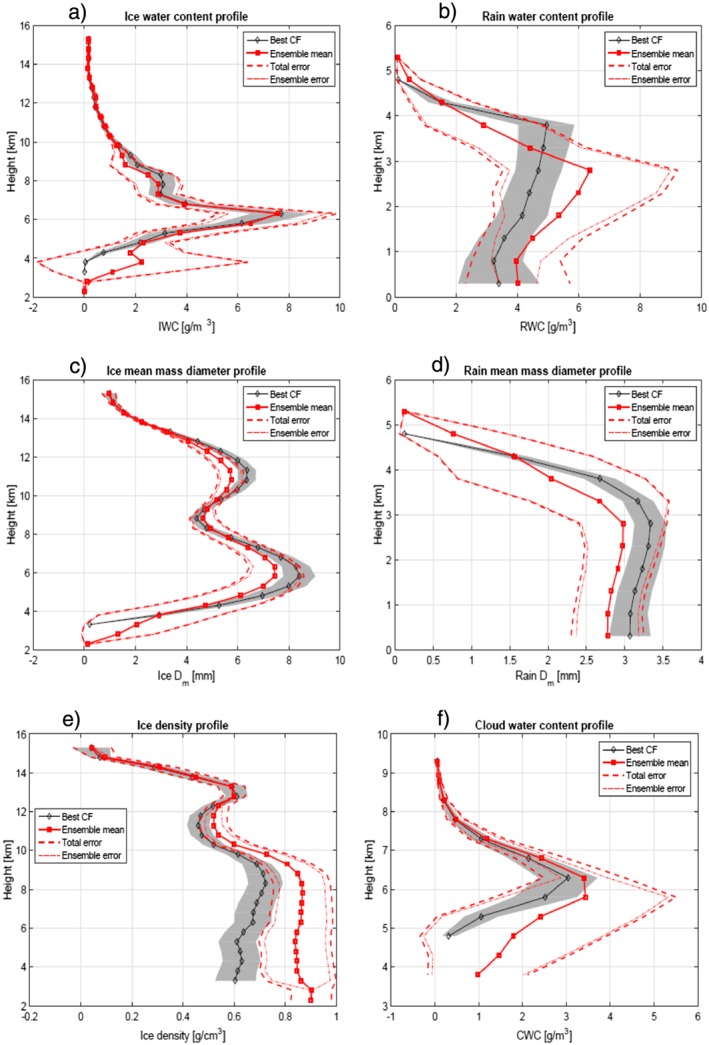
Retrieval of (a, b) ice and rain water contents, (c, d) ice and rain mass‐weighted mean diameter, and (e, f) ice density and cloud water contents for the profile corresponding to LON −80.7425°.

In Figure 12 the retrieval corresponding to the best normalized cost function for measurements ( ${\text{CF}}_{\text{meas}}^{n}$ = 1.8) are depicted with black diamonds with the shaded grey region indicating the retrieval error computed from the covariance matrix of the solution at the last iteration of the retrieval (see equation (5)). The red squares correspond to the ensemble mean computed according to equation (10); the confidence interval for the total error computed by equation (11) is delimited by the dashed lines, while the dash‐dotted lines mark the ensemble error only (second term on the right‐hand side of equation (11)). In regions where this error is dominating the error budget, genuine multiple solutions are possible. This occurs in most of the profile and certainly in the transition region between solid and liquid precipitation, which is not univocally determined by the measurements but is more driven by the a priori selection. On the other hand, where the total error is dominated by the retrieval error of each single profile (e.g., above 13 km), the measurements constrain the retrieval into the same attractor and the uncertainty left in the retrieval parameter space is simply that induced by the measurement and modeling errors.

Figure 11 shows the forward‐modeled reflectivities in correspondence to the solution achieving the best fit to the measurements (diamonds). Measured reflectivities are generally well reproduced, apart from the W band below 9.5 km. This is a common feature in all the retrievals, and it is believed to be related to limitations in the MS modeling, which can be very sensitive to details of the antenna pattern (e.g., presence of side lobes and details of the ice microphysics). Similar issues have been previously reported for Ka band airborne observations [*Battaglia et al.*, 2014]. Modeled and measured (values in parenthesis) BTs and PIAs are also reasonably matched as indicated by the text in the top and bottom left corners.

For this specific profile it is clear that uncertainties are intrinsically large in the bottom part of the atmosphere (below 6 km). In that region only the X band radar is providing useful observations. This is demonstrated in Figure 11 where the forward‐modeled, single‐scattering reflectivities are plotted as dotted lines. Clearly the ranging capabilities of the Ku, Ka, and W band radars are lost below 5, 8, and 9 km, respectively, because of MS effects. Therefore, the retrieval becomes more and more underconstrained when descending down in the troposphere. The retrieval in the rain layer is constrained only by the X band measurements (reflectivities and PIAs). Rain rates at the ground—computed from a standard velocity‐diameter relationship and the retrieved parameters which constrain the PSD—are certainly extreme but with a large level of uncertainty (108 ± 33 mm/h). If the sloping down of the X band profile in the last 4 km close to the surface for a total of roughly 22 dB is interpreted as due to two‐way attenuation then an attenuation coefficient of 2.75 dB/km is predicted. According to *Ryzhkov et al.* [2014] and *Meneghini and Kozu* [1990] at 10°C, X band attenuation is related to the rain rate by RR[mm/h] ≈50(*A*[dB/km])^0.84^ (see their Tables 1 and 4.8, respectively). This would predict a rain rate of 117 mm/h which is in good agreement with our result. Note, on the other hand, that at Ku the anomalous sloping due to MS has an average slope in the 4 km closest to the surface of only 4.5 dB/km, which implies an attenuation of 2.25 dB/km, i.e., smaller than the X band value. If MS effects are ignored and the TRMM relationship RR ≈11.7(*A*[dB/km])^0.65^ is used [*Iguchi et al.*, 2009], then this slope would suggest a rain rate of approximately 54 mm/h.

On the other hand the retrieval seems to consistently predict very large (*D*_*m*_ reaching 8 mm, Figure 12c) , dense (see Figure 12e) particles with large ice contents (exceeding 6 g/m^3^, see Figure 12a) at 6 km altitude. To understand the quality of the retrieval it is useful to plot the averaging kernel matrix **A** (see definition in *Rodgers* [2000]), which can be estimated at iteration *i* as

(12) $$A_{i}\equiv {\hat{S}}_{i}\;J_{i}^{T}\;S_{\varepsilon }^{-1}\;J_{i}.$$

**Figure 13:**
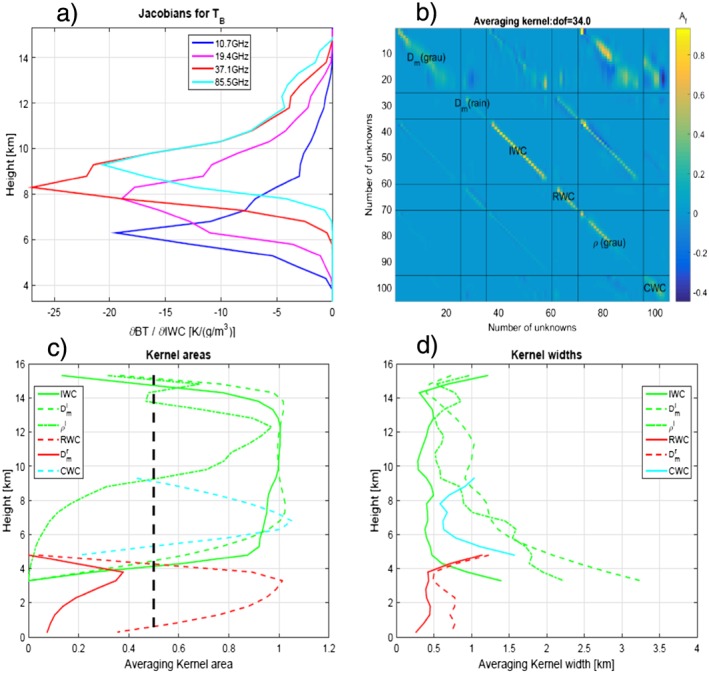
Parameters for diagnosing retrieval performances and measurement information contents for the profile corresponding to LON −80.7425° for the solution which is best fitting the measurements: (a) Jacobian matrix for the four frequency BTs with respect to the ice water content; (b) averaging kernel matrix; and (c) averaging kernel area and (d) width.

The averaging kernel provides the sensitivity of the retrieval to the true state **A** = *∂* **x**_retrieved_/*∂* **x**_true_. An example for the profile shown in Figure 11 is depicted in Figure 13b. In our specific case the state vector represents profiles of different quantities for a total of 105 unknowns. The trace of the averaging kernel matrix provides an estimate of the degrees of freedom for signal, *d* *o* *f*. In this case *d* *o* *f* is equal to 34, i.e., much smaller than the number of unknowns. Thus, our measurements are not providing enough information content on all our retrieved variables. For ideal inversions, **A** would correspond to the unit matrix. Here the matrix **A** is peaked along the main diagonal, but there are areas significantly different from zero in some of the diagonal terms for blocks outside the main diagonal. This is expected because of the interdependence of the retrieval of the different quantities, e.g., of $D_{m}^{i}$, ice water content, and *ρ* ^*i*^. If we restrict our focus to the blocks along the main diagonal, the rows of **A**, here referred to as the “reduced averaging kernel rows,” do typically peak at the right level. Their areas can be considered to be a rough estimate of the fraction of the retrieval that comes from the observations [*Pounder et al.*, 2012]. Values significantly lower than 1 (here we will use 0.5 as a threshold value, dashed black line) highlight regions where the retrieved variables are mainly driven by the a priori. For this profile, as can be seen in Figure 13c, this is certainly the case for $D_{m}^{r}$. This means that all values plotted in Figure 12d are not very significant. On the other hand, the measurements seem to have a greater impact on variables like $D_{m}^{i}$ and IWC, especially at high altitudes.

Following *Rodgers* [2000], but taking the absolute values of the averaging kernel elements to avoid meaningless results, we define an averaging kernel width as

(13) $$W_{A\;i}\equiv {\left[\frac{\underset{j}{\sum }\left|A_{\mathrm{ij}}\right|{\left(H_{i}-H_{j}\right)}^{2}}{\underset{j}{\sum }\left|A_{\mathrm{ij}}\right|}\right]}^{1/2},$$

where *H*_*i*_ is the height corresponding to the retrieved variable and the sums are extended only to the “reduced averaging kernel elements.” This quantity provides an idea about the effective spatial resolution of the retrieval. For instance, the profiles shown in Figure 13d suggest that the retrieval has good spatial resolution (lower than 1 km) apart from regions where the radar capabilities in retrieving the vertical structure are lost (e.g., for the ice phase below 7 km). The radiometer BTs on the other hand provide a much less resolved vertical information as highlighted by the Jacobian with respect to the IWC (Figure 13a), with lower frequencies sounding atmospheric layers closer to the ground. For this profile, however, even the 10.7 GHz channel has maximum sensitivity at about 6 km.

## 4 Retrieval Results

**Figure 14:**
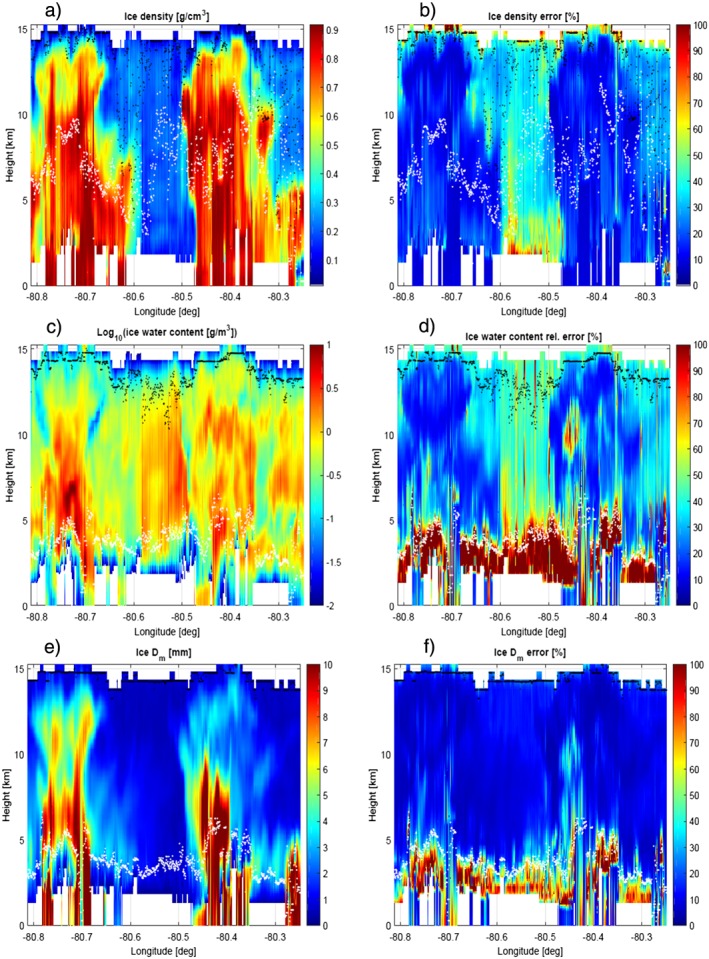
Retrieval of ice density (a), log_10_ of ice water content (c) and ice mass‐weighted mean diameter (e). The region between the white and black dots is where the averaging kernel areas are exceeding 0.5, i.e., where the retrieved variables are mainly driven by the measurements. Retrieval relative errors are shown in the right column (b‐d‐e).

**Figure 15:**
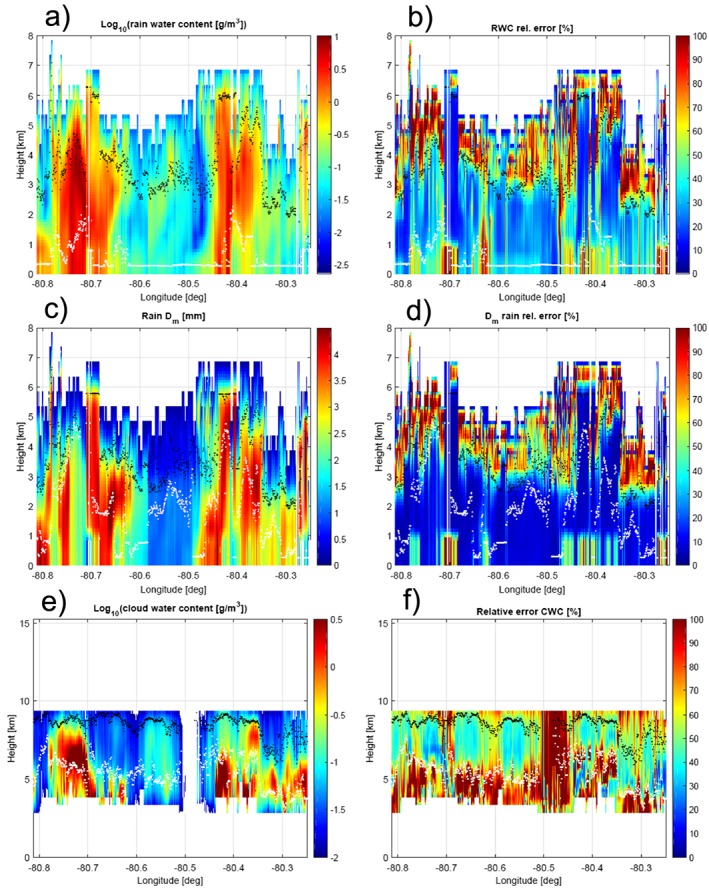
Retrieval of log_10_ of rain water content (a), rain mass‐weighted mean diameter (c) and log_10_ of cloud water content (e). Retrieval relative errors are shown in the right column (b‐d‐e).

The procedure described in the previous sections was applied to the full scene. The results are presented for the ice phase in Figure 14 and for the liquid phase in Figure 15 with the estimated quantities and their corresponding errors in the left and right columns, respectively. All retrieved parameters confirm the convective, extreme nature of the system under observation: (a) ice water contents exceed 10 g/m^3^ within the two cells with integrated ice water paths reaching 25 and 15 kg/m^2^ in the west and east cell, respectively; (b) $D_{m}^{i}$ are above 10 mm both in the west and in the east cell; (c) very large ice densities are retrieved inside the cells with small relative errors in the solution ensemble (Figures 14a and 14b); and (d) rain (cloud) water contents also exceed 8 (6) g/m^3^ and 6 (2) g/m^3^ with integrated rain (cloud) water paths reaching 24 (10) kg/m^2^ and 20 (8) kg/m^2^ in the west and east cells, respectively. All these features are strong evidence of the presence of hail within the cells, as confirmed by the ground‐based radar hydrometeor classification (Figure 7b). The retrieval also suggests the possibility of hail reaching the ground in different areas corresponding to the colored stripes arriving at the surface.

The retrieval provides detailed information particularly in the upper portion of the system where all radar frequencies are not suffering from large attenuation/MS effects. For instance, high‐density ice particles exceeding mass‐weighted mean diameters of 2–3 mm (cyan colors) are found above 13 km in both cells. Ice PSDs with mass‐weighted mean diameters in that range are the most efficient scatterers at W band and are capable of producing W band attenuation in excess of 5 dB/km/(g/m^3^) (see cyan lines in Figure 8b). The increased levels of attenuation inside the convective cells are highlighted by the continuous, dashed, and dotted green lines drawn onto the W band measured reflectivity (Figure 5d). These lines represent the levels—as computed from the retrieved solution ensemble—at which the top‐down optical thickness exceeds 1, 3, and 5 (i.e., two‐way attenuations of 8.7, 26, and 43.4 dB).

Similarly, ice PSDs with *D*_*m*_ in the range between 4 and 9 mm are the most efficient scatterers for Ka band radiation (red lines in Figure 8). $D_{m}^{i}$ in this range is retrieved in correspondence to the strong Ka‐attenuating region at heights of about 8–11 km (6–9 km) in the west (east) cell (compare Figure 5c with Figure 14e). The fact that the west cell is characterized by the presence of larger particles in the layer above 8–9 km compared to the east cell also explains the larger BT depressions observed at 85.5 GHz within the east cell (see Figure 10): in fact, for the same optical thickness, smaller particles are more effective in scattering radiation backward (because of lower asymmetry parameter, *g*, see Figure 6 in *Battaglia et al.* [2015]) and thus to preferentially favor scattering of cold cosmic‐originated radiation (more than warm surface‐originated radiation) into the radiometer field of view [*Battaglia et al.*, 2005]. Note that as a rule of thumb we do expect that the so‐called generalized weighting functions [see *Mugnai et al.*, 1993] are peaked certainly within the region with top‐down optical thickness lower than 3, which can be deduced from the plot for the closely behaved 94 GHz frequency (green lines in Figure 5d).

The retrieved results can be forward modeled to compute single and multiple scattering reflectivities; thus, they can be exploited to assess the importance of MS for the different channels. The levels where the MS enhancement exceeds 6 dB for the four radar frequencies are plotted in Figure 5a (blue, green, black, and cyan lines corresponding to X, Ku, Ka, and W band, respectively). While some MS enhancement is present at X band it rarely exceeds 6 dB (and only in the west cell). This supports the idea that the X band is the only channel really capable of fully penetrating the storm in the centers of the two deep convective cores, while the Ku band is heavily affected by MS in the same regions. As a result the Ku ranging capabilities are drastically, if not completely, reduced below 6 and 5 km for the west and east cells, respectively. The relevance of MS for the ER‐2 Ku radar, which has a footprint at the ground slightly exceeding 1 km, demonstrates that such an effect is certainly not negligible for Ku band spaceborne systems like those on board the TRMM and GPM satellites, which are characterized by footprints almost 5 times bigger. This confirms previous findings based on theoretical computations [*Battaglia et al.*, 2006] and on other airborne/GPM observations [*Battaglia et al.*, 2014, 2016].

As expected, Ka and W bands are heavily affected by MS, but with distinct behaviors in the two cells: While in the west cell the Ka and W signals behave similarly with a penetration down to only approximately 9 km, in the east cell the W band signal is penetrating only down to 10–11 km, while the Ka reaches below 6 km. This is mirrored by the behavior of the BTs for the 37 and 85.5 GHz channels and, as explained before, is believed to be linked to the presence of a large amount of dense ice particles with $D_{m}^{i}$ of the order of 0.5–3 mm in the east cell. Incidentally, the mean Doppler velocities appear very noisy in correspondence to the areas characterized by strong MS enhancement, even if the signal is significantly above noise (not shown). This is connected to a decorrelation effect of MS, which is known to broaden the Doppler spectrum [e.g., *Battaglia et al.*, 2013]. Further investigations of this aspect are left for future work.

### 4.1 Errors

**Figure 16:**
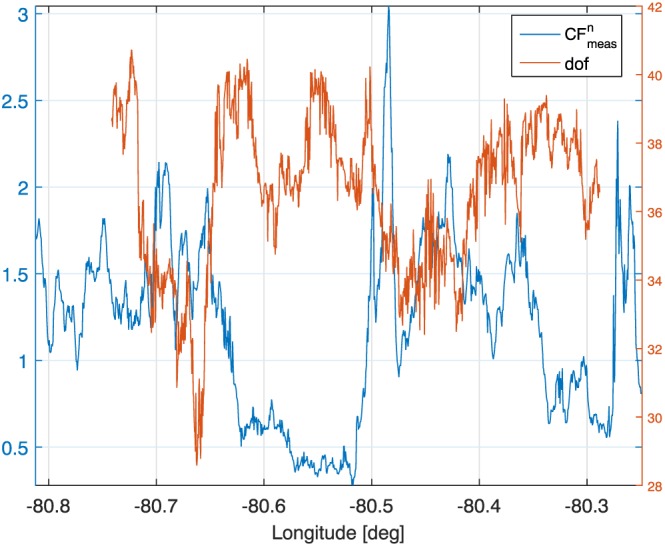
Normalized cost function and degree of freedom.

Between 30 and 40° of freedom of signal are associated with the current suite of measurements (see Figure 16) and are thus insufficient to provide full microphysics profiling. Only some of the retrieved parameters are therefore genuinely characterized by the measurement vector. The ice mass‐weighted mean diameters (Figure 14e) are the quantities that are undoubtedly retrieved with more confidence. First, they exhibit the highest information content coming from measurements. This is highlighted by the white and black dots that identify, respectively, the lower and upper boundaries of the region where the areas of the averaging kernels exceed 0.5. Second, errors in $D_{m}^{i}$ (Figure 14f) are generally small (lower than 20%) in the upper regions of the system. This is expected because, in absence of attenuation and MS effects, DWRs provide a direct information of the size of the particles within the backscattering volume, with the three different pairs of DWRs being more effective at different altitudes and for different range of sizes (Figure 8a). The large errors in the solid‐liquid transition zone are mainly driven by a large variability in the ensemble of solutions (second term in the square root of equation (11)), which reflects the uncertainties in the identification of the transition between the solid and liquid phase.

Ice water contents are also mostly driven by measurements as highlighted by the fact that the region between the white and black dots encompasses most of the ice region. On the other hand, the retrieved ice density has information mainly coming from the a priori density profile (in Figures 14a and 14b only the white and black dots correspond to the lower and upper boundaries of the region where the areas of the averaging kernels exceed 0.3). Despite this, the errors on densities remain relatively small in relative sense in the core of the convective cores because the ensemble errors are small, while they strongly increase in between the two cells. As a result uncertainties in IWC also strongly increase in such region.

The liquid phase (Figure 15) is certainly more challenging with large uncertainties inside the cells due to the reduced profiling capability of the ER‐2 suite of sensors. Uncertainties and information contents significantly increase in between the two cells where all radar frequencies are effectively penetrating down to the surface.

### 4.2 Quality of the Retrieval

Overall, when forward modeled, the retrieved profiles well reproduce the measurements with ${\text{CF}}_{\text{meas}}^{n}$ values typically smaller than 1.5 (Figure 16). Mean values and standard deviations of the reflectivity residuals are plotted in Figure 10a as a function of the X band reflectivity. The three lowest frequencies exhibit residuals in line with the assumed measurement errors; on the other hand, the W band residuals seem to be slightly negatively biased and have standard deviations increasing at larger X band reflectivities. This can be due respectively to a calibration issue (high‐biased reflectivities) and to the aforementioned issue in the ice and MS modeling. Reflectivity residuals are generally higher in regions with stronger spatial variability. This is likely due to the mismatching of the radar backscattering volumes at the different frequencies. Similarly, brightness temperatures and PIAs are also generally well fitted (center and bottom panels) with larger residuals typically found at the edges of the convective cores.

## 5 Summary and Conclusions

The characterization of hydrometeor microphysical properties (size, mass, and density) in severe convective systems remains a thorny problem. This study presents a methodology for retrieving hydrometeor profiles of ice, rain, and cloud from a unique and unprecedented suite of active and passive microwave airborne observations, which provide top‐down information that are very complementary to more conventional ground‐based measurements.

The retrieval, based on optimal estimation, is applied to two convective cells observed on 23–24 May 2014 over North Carolina during the NASA IPHEx campaign. The key findings of our study can be summarized as following.

1. In deep convective systems multiwavelength airborne radar observations are the result of a complex interplay between attenuation, non‐Rayleigh, and MS effects. For a correct interpretation of the radar signal all these effects must be properly accounted for in the radar forward modeling. The large impact of attenuation and MS at the higher frequencies caused by high‐density ice at high altitudes significantly reduces the profiling capabilities of a multifrequency suite of radars when going deep into convective cores. For the North Carolina 23–24 May ER‐2 observations, the four‐frequency profiling system is effectively reduced to a single‐frequency (X band) one at the center of the convective cores in proximity to the surface. In trailing stratiform precipitation, on the other hand, the full suite of radars may be effective down to the surface.
2. Optimal estimation algorithms are powerful tools for understanding deep convective systems by shedding light onto vertical profiles of cloud microphysics (e.g., profiles of ice water content, ice density, and ice mass‐weighted mean diameter). Because of their complexity, a variety of a priori assumptions (like those related to the maximum density of ice particles in the profile or the height of the melting region) must be explored; the solution space can then be significantly narrowed down by selecting only those solutions that are best matched with the measurements. In this ensemble approach the spread of the best converging solutions provides a measure of the accuracy of the retrieved variables.
3. For the convective scenes here considered between 30 and 40 degrees of freedom of signal are associated to the current suite of measurements and are insufficient to provide full microphysics profiling. For the ice segment of the cloud, measurements have the largest impact onto the retrieval of characteristic sizes, followed by equivalent water contents. Ice densities are more driven by the a priori assumptions. Still, uncertainties in ice density are relatively small in extensive regions of the convective system, an indication that only certain densities can explain the observations.
4. In the core of convective cells, one‐way PIA can reach 20 dB at X band. This suggests values larger than 40 dB at Ku band. Part of this attenuation can be attributed to dense ice particles aloft, which are characterized by very high single‐scattering albedo and thus produce a very efficient source of MS. MS is playing an important role in affecting the signal at radar frequencies above the X band and must be accounted for in order to correctly interpret the observations. This has strong implications for satellite retrievals. For instance, the estimates of precipitation as derived from the Tropical Rainfall Measuring Mission Precipitation Radar (and similarly for the GPM Ku band radar) signal by current algorithms (which do not account for MS) must be taken with extreme caution in presence of dense, ice‐laden convective cells.

This work provides a first step toward remote sensing microphysical characterization of deep convective clouds by a multifrequency suite of active and passive microwave instrument observations. Future work will aim (1) at refining the retrieval algorithm along the following guidelines: (a) better definition of a priori ensemble profiles (e.g., by using the Doppler information); (b) introduction of correlations between different microphysical parameters; (c) inclusion of melting hydrometeors and of more sophisticated scattering properties for nonspherical ice crystals at the high‐frequency channels; and (d) extending the observation vectors to Doppler and dual‐Doppler variables; (2) at quantifying the information content of the different measurements (e.g., that provided by the BTs); and (3) at deriving a climatology of convective cloud microphysics by applying the algorithm to the relevant cases observed with the ER‐2 suite of instruments and by including ground‐based remote sensing observations into the measurement vector. This could then be exploited in better understanding ice processes and in improving the microphysics in spaceborne algorithms.

## Supporting information

Supporting Information S1
